# A comparison of physical activity, muscle strength, and sleep between people with type 2 diabetes in Kuwait and the UK: A cross-sectional study

**DOI:** 10.3389/fendo.2022.1067227

**Published:** 2022-12-08

**Authors:** Ebaa Al Ozairi, Dalal Alsaeed, Dherar Al Roudhan, Nia Voase, Jill P. Pell, Frederick K. Ho, Mohammed Abdulla, Stuart R. Gray

**Affiliations:** ^1^ Clinical Research Unit, Dasman Diabetes Institute, Kuwait City, Kuwait; ^2^ Department of Medicine, Faculty of Medicine, Kuwait University, Kuwait City, Kuwait; ^3^ School of Health and Wellbeing, University of Glasgow, Glasgow, United Kingdom; ^4^ School of Cardiovascular and Metabolic Health, University of Glasgow, Glasgow, United Kingdom

**Keywords:** cross-sectional study, diabetes mellitus, type 2, muscle strength, Kuwait, United Kingdom

## Abstract

**Background:**

The aim of the current study was to determine the prevalence of low muscle strength and to evaluate physical activity and sleep characteristics in people with type 2 diabetes in Kuwait. Additionally, equivalent data from the UK Biobank cohort were compared.

**Methods:**

People with type 2 diabetes from the UK Biobank (n = 23,570) and Kuwaiti cohorts (n = 3,135) were included in this cross-sectional study. Self-reported sleep, physical activity, and muscle strength were compared between the cohorts, using linear and logistic regression, with adjustments for age, sex, and duration of diabetes.

**Results:**

Physical activity levels (−1216 (−1328,1104 Met-min/wee k: standardized B-coefficient −0.52 (−0.57, −0.47) and grip strength (−3.2 (−3.58, −2.82) kg: standardized B-coefficient (−0.29 (−0.32, -0.26) were lower in the Kuwaiti cohort and the odds of having short sleep (OR 1.32 (1.19,1.46), being classed as inactive (OR 8.70 (7.59, 9.98) and having muscle weakness were higher (OR 1.88 (1.69, 2.09). These analyses were adjusted for age, sex, and duration of diabetes.

**Conclusions:**

This study demonstrates that insufficient sleep, physical inactivity, and muscle weakness are prevalent in people with type 2 diabetes, especially in Kuwait. Importantly, these observations warrant urgent and effective interventions to improve sleep, muscle strength, and physical activity, especially in Kuwait.

## Introduction

Currently, 537 million adults (20–79 years) are living with diabetes worldwide—a global prevalence rate of 10%—and it is predicted that this number will increase to 643 million by 2030 and to 784 million by 2045 (1). Furthermore, in 2021, 6.7 million deaths were attributed to diabetes (1). Among diabetics, approximately 90%–95% have type 2 diabetes, which is associated with a greater risk of not only microvascular complications, such as neuropathy, nephropathy, and retinopathy, but also macrovascular sequelae such as coronary artery disease, peripheral arterial disease, and stroke (2, 3). Indeed, people with type 2 diabetes have a two- to threefold greater risk of cardiovascular disease (CVD), and approximately 80% of all deaths among individuals with type 2 diabetes are due to CVD (4). Type 2 diabetes is clearly a worldwide problem, but it is of particular concern in countries like Kuwait where the standardized prevalence of diabetes in adults (20–79 years) is estimated to be 24.9% (1).

Apart from the complications associated with type 2 diabetes, potentially bidirectional effects on skeletal muscle strength and physical capabilities have also been described. Indeed, type 2 diabetes results in accelerated loss of muscle strength (5, 6), and low muscle strength is associated with poorer glycemic control and increased risk of complications (7). Additionally, such low muscle strength in people with type 2 diabetes can result in impaired physical function, loss of independence, and, ultimately, lower quality of life (8–10). Strategies for the management of type 2 diabetes and its complications are therefore crucial, and lifestyle factors are key modifiable determinants. Physical activity can be beneficial in people with type 2 diabetes (11, 12), and emerging evidence indicates that sleep may also be critical, with both duration and quality being important (13, 14).

Differences exist in the prevalence of physical inactivity, poor sleep, and low muscle strength between populations of the world, and while data on these variables are available for several countries, scant data from Kuwait indicate low levels of physical activity and poor sleep habits (15, 16). Importantly, no data on physical function are currently available in Kuwait. Together, such information will enable detailed characterization of physical function, physical activity, and sleep in people with type 2 diabetes in Kuwait. Additionally, a comparison with congruent data from the UK will greatly facilitate the development of appropriate lifestyle interventions. Therefore, the aim of the current study was to determine the prevalence of low muscle strength and to evaluate physical activity and sleep characteristics in people with type 2 diabetes in Kuwait. Additionally, equivalent data from the UK Biobank cohort were compared.

## Methods

To obtain data for the Kuwaiti population, we recruited 3,135 patients with type 2 diabetes from diabetes clinics at the DASMAN Diabetes Institute in Kuwait City, Kuwait, from October 2019 to January 2022 inclusive. The study obtained ethical approval from the DASMAN Office for Regulator Affairs ethical committee, and all participants provided written informed consent. For comparison, we included UK Biobank data from 23,570 individuals with type 2 diabetes at recruitment. Type 2 diabetes was ascertained based on the nurse-led interview at baseline. Diabetes diagnosed after the age of 30 years was defined as type 2 diabetes. The UK Biobank Study was approved by the NorthWest MultiCentre Research Ethics Committee (Ref 11/NW/0382 on 17 June 2011), and all participants provided written informed consent. This study was conducted using the UK Biobank resource, under application number 7155.

### Procedures

The procedures for data collection were the same in the UK Biobank and Kuwaiti cohorts. Data on demographic (age at recruitment and sex), lifestyle (smoking status), and health (medical history and duration of diabetes) factors and measurements were collected for both cohorts. Measurements included height, weight, waist circumference, and blood pressure. Grip strength for the right and left hands was measured in both cohorts using the Jamar grip strength dynamometer, and the average of these readings was used for analysis. Low muscle strength was defined as a low grip strength of <27 kg in men or <16 kg in women, in accordance with the guidelines issued by the European Working Group Sarcopenia in Older People (EWGSOP2) (17). Physical activity was self-reported using the short-form International Physical Activity Questionnaire (IPAQ), and activity lower than 500 MET-min/week was defined as inactive. Sleep duration was self-reported, but participants were also asked questions on 1) ease of getting up in the morning, 2) whether they considered themselves to be a morning/evening person, 3) napping habits, 4) trouble falling asleep at night or waking up in the middle of the night, and 5) likelihood of unintentionally falling asleep during the day. Blood samples were collected and analyzed for HbA1c, lipid profile, vitamin D, and kidney and liver function using standard biochemical methods.

### Statistical analyses

Participant characteristics are presented separately for the two cohorts with continuous variables summarized as means (SD) and categorical variables as frequencies (%) with comparisons between UK and Kuwait *via* univariable linear regression with country variable (UK or Kuwait) as the exposure. Multivariable linear regression analyses were used for continuous outcomes, and logistic regression was applied for binary outcomes to explore differences between UK and Kuwaiti participants. The country variable (UK or Kuwait) was the primary exposure of interest. Continuous outcomes include MET-minutes from walking, moderate physical activity (MPA), vigorous physical activity (VPA), and all physical activity (PA); body mass index (BMI); waist circumference; and grip strength. Binary outcome variables include physical inactive; sleep < 7 h; usually nap; and usually sleepiness, often dozing, evening chronotype, and low grip strength. Age, sex, and duration of diabetes were adjusted for in the main model (model 1). Two additional models were constructed to analyze muscle strength outcomes, and these were additionally adjusted for physical activity (total physical activity), sleep, and adiposity markers (waist circumference and BMI), as they may explain potential differences in muscle strength. Results are represented as regression coefficients standardized to their SD for continuous outcomes and as standardized odds ratios for binary outcomes for model 1 and presented as forest plots. A two-tailed 5% statistical significance was used so that all associations with 95% confidence intervals that did not overlap with the null value could be labeled significant.

## Results

Descriptive characteristics for the UK Biobank and the Kuwaiti cohorts are listed in **Table 1**. The Kuwaiti cohort had a higher percentage of men, had suffered from diabetes for a longer period in spite of being younger, had lower BMI and waist circumference, and had higher HbA1c concentrations but lower triglycerides and total cholesterol. Sleep, physical activity, and grip strength data are presented in **Table 2** with unadjusted comparison p-values presented. Kuwaitis were more likely to sleep <7 h and to nap and less likely to be sleepless, doze, and have an evening chronotype. Kuwaitis report lower walking, moderate, vigorous, and total physical activities and were more likely to be classified as inactive. Grip strength was lower in the Kuwaitis, and they were more likely to be classified as having low grip strength.

**Table 1:** Characteristics of the UK Biobank and Kuwaiti cohorts of people with type 2 diabetes.

Characteristic	UK Biobank (n = 23,570)	Kuwait (n = 3,135)	p-Value
Age (years)	60.1 (6.8)	54.1 (18.0)	<0.0001
Male*	8,864 (38%)	1,568 (50%)	<0.0001
Female*	14,706 (62%)	1,564 (50%)	
Duration of diabetes (years)	6.7 (6.2)	17.3 (9.6)	<0.0001
BMI (kg/m^2^)	31.6 (5.9)	30.8 (13.9)	<0.0001
Waist circumference (cm)	103.6 (14.1)	99.2 (16.4)	<0.0001
Total cholesterol (mmol/L)	4.5 (1.0)	4.2 (2.4)	<0.0001
Triglycerides (mmol/L)	2.2 (1.3)	1.4 (4.4)	<0.0001
HbA1c (mmol/mol)	53.0 (13.8)	62.1 (16.7)	<0.0001

Numbers presented are mean (SD) unless denoted with *, which are n (%).

BMI, body mass index.

**Table 2:** Physical activity, sleep, and physical function data in UK Biobank and Kuwaiti cohorts of people with type 2 diabetes.

Characteristic	UK Biobank (n=23,570)	Kuwait (n=3,135)	p-value
Sleep < 7h*	6,626 (28%)	1,141 (36%)	< 0.0001
Usually nap*	2,778 (12%)	752 (24%)	< 0.0001
Usually sleepless*	8,481 (36%)	408 (13%)	< 0.0001
Often dozing*	8,888 (38%)	863 (28%)	< 0.0001
Evening chronotype*	8,084 (34%)	611 (19%)	< 0.0001
Walking (MET-min)	933.4 (1,046.8)	623.6 (856.0)	< 0.0001
Moderate Physical Activity (MET-min)	852.5 (1,237.5)	143.1 (549.3)	< 0.0001
Vigorous Physical Activity (MET-min)	521.6 (1,205.8)	153.9 (703.0)	< 0.0001
Total Physical Activity (MET-min)	2,252.9 (2,386.0)	920.5 (1,353.5)	< 0.0001
Inactive*	8,620 (37%)	1,879 (85%)	< 0.0001
Grip strength	30.0 (11.0)	24.8 (10.4)	< 0.0001
Low grip strength*	4,135 (18%)	984 (33%)	< 0.0001

Numbers presented are mean (SD) unless denoted with *, which are n (%).

**Figure 1:**
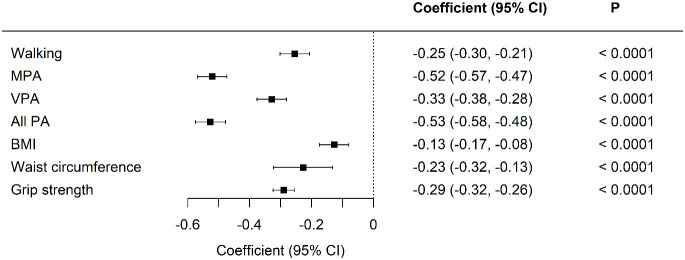
Standardized differences in physical activity, body composition, and grip strength between UK Biobank and Kuwaiti cohorts of people with type 2 diabetes A negative coefficient means a lower value in the Kuwaiti cohort. MET-minutes in MPA, moderate physical activity; VPA, vigorous physical activity; PA, physical activity; BMI, body mass index. Adjusted for age, sex, and duration of diabetes.

**Figure 2:**
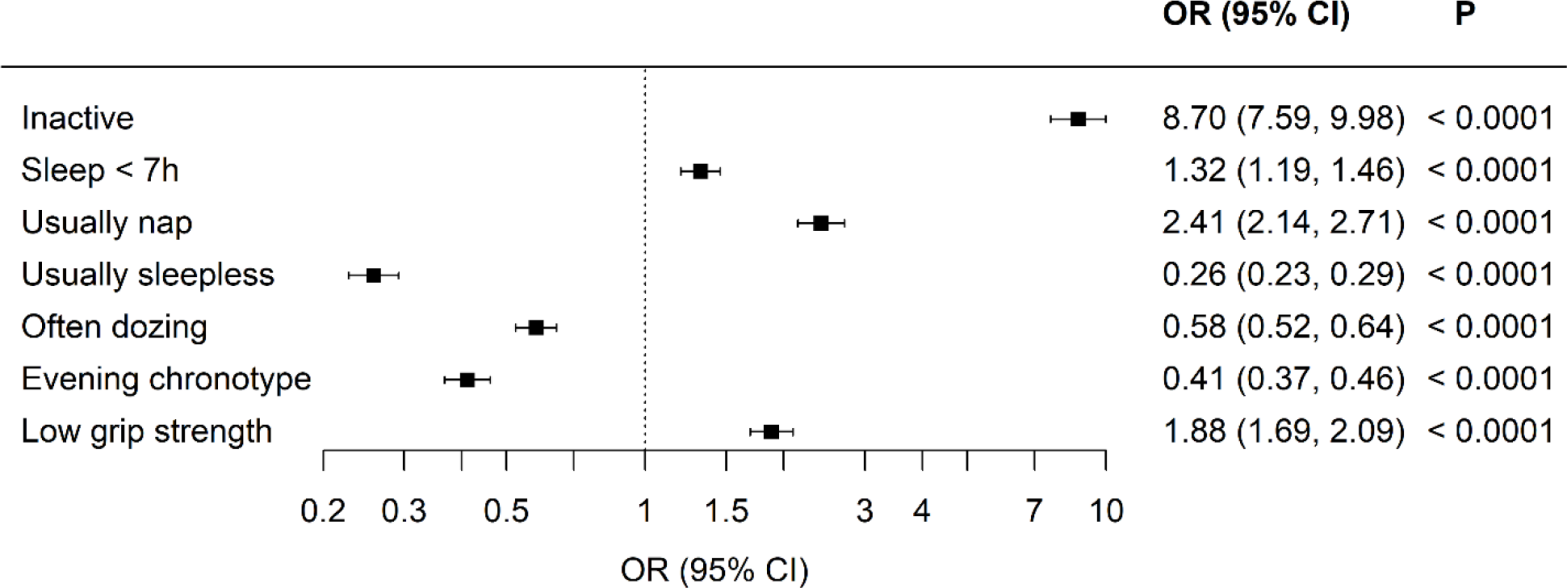
Odds ratios of physical activity, sleep, and strength characteristics comparing the Kuwaiti cohort to the UK Biobank of people with type 2 diabetes, An odds ratio lower than 1 means lower odds in the Kuwaiti cohort. Adjusted for age, sex, and duration of diabetes.

Standardized differences between the two cohorts were visualized in model 1, with adjustments for age, sex, and duration of diabetes (**Figures 1** and **2**). All aspects of physical activity were lower in the Kuwaiti cohort, including grip strength and moderate-intensity physical activity (**Figure 1**). The Kuwaiti participants had greater odds of being defined as inactive (<500 MET·min^−1^·week^−1^) and having insufficient sleep (<7 h/night) and muscle weakness (low grip strength) (**Figure 2**). Kuwaitis were also more likely to nap but less likely to report dozing, being sleepless, or having an evening chronotype (**Figure 2**). Conversely, BMI and waist circumference were lower in the Kuwaiti cohort (**Figure 1**). Adjustment of PA, sleep, and markers of adiposity (BMI and waist circumference) did not attenuate the differences in grip strength between the UK and Kuwait cohorts (**Table 3**).

**Table 3:** Muscle strength outcomes by adjustment models.

	Model 1		Model 2		Model 3	
	Coefficient/OR (95% CI)	p	Coefficient/OR (95% CI)	p	Coefficient/OR (95% CI)	p
Grip strength	−3.20 (−3.58, −2.82)	<0.0001	−3.47 (−3.91, −3.03)	<0.0001	−3.97 (−4.88, −3.07)	<0.0001
Low grip strength	1.88 (1.69–2.09)	<0.0001	2.05 (1.79–2.34)	<0.0001	2.01 (1.53–2.63)	<0.0001

Model 1: adjusted for age, sex, and duration of diabetes. Model 2: additionally adjusted for total PA and sleep. Model 3: additionally adjusted for BMI and WC.

PA, physical activity; BMI, body mass index; WC, waist circumference.

## Discussion

The results of the current study demonstrated that, compared to the UK Biobank cohort, the Kuwaiti cohort of type 2 diabetics is more likely to report insufficient sleep, physical inactivity, and muscle weakness. Specifically, while the prevalence of insufficient sleep was only slightly higher, that of muscle weakness and physical inactivity was alarmingly high, implying that interventions to improve sleep, muscle strength, and physical activity are especially important for people with type 2 diabetes in Kuwait. Other interesting differences noted were the longer duration of diabetes and poorer glycemic control in people with type 2 diabetes in Kuwait, even though they were younger and less obese. Much of the differences in lifestyle observed here may be due to many differences between the countries, such as the extremely warm weather, low walkability, food and physical activity culture, and relative accessibility and ownership of motor vehicles in Kuwait.

The prevalence of insufficient sleep is high worldwide; for example, data from the National Health and Nutrition Examination Survey in the USA report a prevalence of ~37% (18), which is similar to our data from Kuwait. Such prevalence of insufficient sleep is concerning because it is associated with a greater risk of a broad range of health outcomes, including mortality, hypertension, cardiovascular diseases, obesity, metabolic syndrome, and type 2 diabetes (18–21). There is, therefore, a broad need for interventions that will improve sleep habits, especially in individuals with type 2 diabetes, because the risk of poor health outcomes is higher. Effective psychological and behavioral interventions to improve sleep have been described, such as sleep hygiene and cognitive behavioral therapy (22), and there is some evidence of their effectiveness in people with type 2 diabetes (23). Even though this is encouraging, it will be necessary to develop, adapt, test, and implement such interventions in people with type 2 diabetes, both in the UK and in Kuwait, because the causes of poor sleep will not be identical among populations from different countries.

There is also some evidence that physical activity, among the multitude of other benefits (24), can also help improve sleep habits (25). Moreover, physical activity is recommended for people with type 2 diabetes because it can improve blood glucose control and reduce the risk of comorbidities such as hyperlipidemia, hypertension, and ischemic heart disease (26, 27). Despite data on the prevalence of physical inactivity in the adult UK population (28), there is no such information for the Kuwaiti population. A recent meta-analysis has estimated that 63.5% of the general Kuwaiti population does not meet current World Health Organization recommendations of 150 min/week of moderate-intensity physical activity (e.g., 29); however, it was noted that data were limited (15). Although the current study used a marginally different definition of being inactive, our data show that inactivity is even higher among a slightly older sample of people with type 2 diabetes. Such high levels of inactivity are alarming and represent an immediate need for intervention. While, to the best of our knowledge, there are no physical activity interventions in people with type 2 diabetes in Kuwait, a recent meta-analysis of interventions to promote physical activity among adults and children across Gulf countries found that interventions encouraging step counting and walking increased daily step count; however, the number of studies was limited. However, multicomponent interventions, including physical activity that targeted obesity and people with type 2 diabetes, did not increase physical activity (30). Thus, interventions developed in other countries, such as the UK, are unlikely to be directly transferable due to differences in factors such as climate, transport policy, social traditions, and physical activity infrastructure. Hence, tailored multilevel interventions that are locally effective need to be developed.

Physical inactivity is much higher in the Kuwaiti population than in the UK population, and low levels of physical activity also probably contribute to the prevalence of muscle weakness (31). It is known that type 2 diabetes accelerates the loss of muscle mass with age, thereby resulting in a higher prevalence of muscle weakness in people with type 2 diabetes; however, the prevalence of muscle weakness in the Kuwaiti cohort was much higher than that reported in the UK and other countries (5, 6, 32, 33). As muscle weakness is associated with poor health outcomes, low quality of life, falls, and loss of independence and has vast economic costs (34–37), interventions to improve muscle strength are warranted (38).

The above notwithstanding, this study has a few limitations. First, the representativeness of both cohorts is a potential issue because, in Kuwait, the cohort was recruited from a tertiary diabetes care center, which may not reflect the wider Kuwaiti population with type 2 diabetes. Similarly, the UK Biobank cohort has been shown to be not representative of the UK population in that it suffers from a healthy volunteer bias (39). Although data for socioeconomic status were available for the UK Biobank cohort, no information was available for the Kuwaiti cohort, and this may be a potential confounder that we are not able to account for. While both cohorts were different in basic characteristics, such as age and duration of diabetes, these were adjusted for in our analysis and likely represent the different phenotypes of type 2 diabetes in Kuwait. Nonetheless, the magnitude of differences between these cohorts, in terms of physical activity and muscle strength, is unlikely to be due to these differences.

In summary, this study demonstrates that insufficient sleep, physical inactivity, and muscle weakness are prevalent in people with type 2 diabetes, especially in Kuwait. Importantly, these observations warrant urgent and effective interventions to improve sleep, muscle strength, and physical activity, especially in Kuwait.

## Data availability statement

The raw data supporting the conclusions of this article will be made available by the authors, without undue reservation.

## Ethics statement

The studies involving human participants were reviewed and approved by DASMAN Office for Regulator Affairs ethical committee. The patients/participants provided their written informed consent to participate in this study.

## Author contributions

EA, DAS, FH, MA and SG were responsible for the conceptualization of the study. SG and FH performed the analysis of the data. EA, DAS, DAR, NV, MA, JPP, and FH were responsible for data collection and curation. SG, FH, and EA were responsible for writing the first draft, and all other authors were responsible for reviewing and editing the manuscript. All authors contributed to the article and approved the submitted version.
