# Corrigendum: A comparison of physical activity, muscle strength, and sleep between people with type 2 diabetes in Kuwait and he UK: a cross sectional study

**DOI:** 10.3389/fendo.2023.1278604

**Published:** 2023-10-09

**Authors:** Ebaa Al Ozairi, Dalal Alsaeed, Dherar Al Roudhan, Nia Voase, Jill P. Pell, Frederick K. Ho, Mohammed Abdulla, Stuart R. Gray

**Affiliations:** ^1^ Clinical Research Unit, Dasman Diabetes Institute, Kuwait City, Kuwait; ^2^ Department of Medicine, Faculty of Medicine, Kuwait University, Kuwait City, Kuwait; ^3^ School of Health and Wellbeing, University of Glasgow, Glasgow, United Kingdom; ^4^ School of Cardiovascular and Metabolic Health, University of Glasgow, Glasgow, United Kingdom

**Keywords:** cross-sectional study, diabetes mellitus, type 2, muscle strength, Kuwait, United Kingdom

In the published article, as a result of an error in some of the calculations of physical activity variables, there were mistakes in **Figure 1** and **Table 2** as published. The corrected **Figure 1**, **Table 2**, and their captions appear below.

**Table 2:** Physical activity, sleep and physical function data in UK Biobank and Kuwaiti cohorts of people with type 2 diabetes.

Characteristic	UK Biobank (n=23,570)	Kuwait (n=3,135)	p-value
Sleep < 7h*	6,626 (28%)	1,141 (36%)	< 0.0001
Usually nap*	2,778 (12%)	752 (24%)	< 0.0001
Usually sleepless*	8,481 (36%)	408 (13%)	< 0.0001
Often dozing*	8,888 (38%)	863 (28%)	< 0.0001
Evening chronotype*	8,084 (34%)	611 (19%)	< 0.0001
Walking (MET-min)	933.4 (1,046.8)	623.6 (856.0)	< 0.0001
Moderate Physical Activity (MET-min)	852.5 (1,237.5)	143.1 (549.3)	< 0.0001
Vigorous Physical Activity (MET-min)	521.6 (1,205.8)	153.9 (703.0)	< 0.0001
Total Physical Activity (MET-min)	2,252.9 (2,386.0)	920.5 (1,353.5)	< 0.0001
Inactive*	8,620 (37%)	1,879 (85%)	< 0.0001
Grip strength	30.0 (11.0)	24.8 (10.4)	< 0.0001
Low grip strength*	4,135 (18%)	984 (33%)	< 0.0001

**Figure 1:**
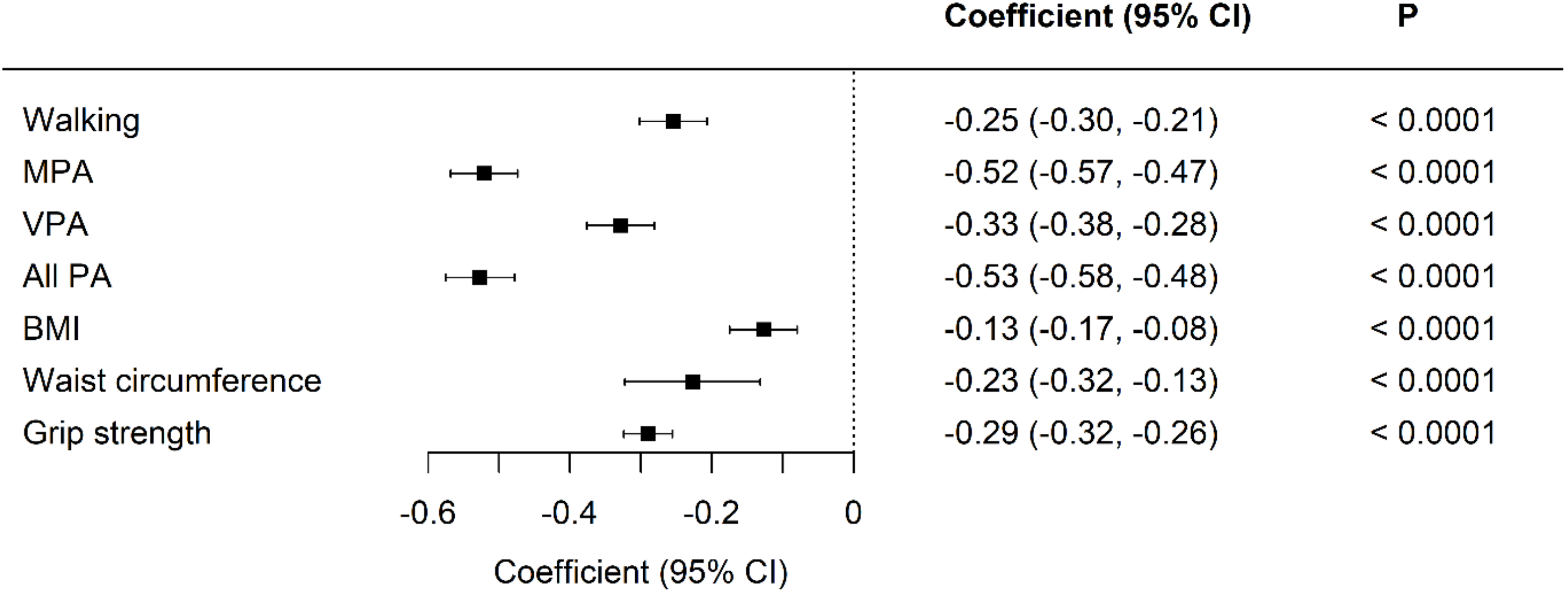
Standardized differences in physical activity, body composition, and grip strength between UK Biobank and Kuwaiti cohorts of people with type 2 diabetes A negative coefficient means a lower value in the Kuwaiti cohort. MET-minutes in MPA, moderate physical activity; VPA, vigorous physical activity; PA, physical activity; BMI, body mass index. Adjusted for age, sex, and duration of diabetes.

Furthermore, because of an error in some of the calculations of physical activity variables, there were some mistakes in the body text in article as published. A correction has been made to **[Abstract]**, [*Results* **]**, [Paragraph 1]. This sentence previously stated:

“Physical activity levels (−937 (−1,097, −851) Met-min/week: standardized B-coefficient −0.42 (−0.47, −0.37)) and grip strength (3.2 (−3.58, −2.82) kg: standardized B-coefficient (−0.29 (−0.32, −0.26)) were lower in the Kuwaiti cohort, and the odds of having short sleep (OR 1.32 (1.19,1.46), being classed as inactive (OR 8.70 (7.59, 9.98), and having muscle weakness (OR 1.88 (1.69, 2.09) were higher.”

The corrected sentence appears below:

“Physical activity levels (-1216(-1328,1104 Met-min/wee k: standardized B-coefficient -0.52 (-0.57, -0.47) and grip strength (-3.2(-3.58, -2.82) kg: standardized B-coefficient (-0.29 (-0.32, -0.26) were lower in the Kuwaiti cohort and the odds of having short sleep (OR 1.32 (1.19,1.46), being classed as inactive (OR 8.70 (7.59, 9.98) and having muscle weakness were higher (OR 1.88 (1.69, 2.09).”

In addition, a further correction has been made to **[Abstract]**, [*Conclusions* **]**, [Paragraph 1]. This sentence previously stated:

“The aim of the current study was to determine the prevalence of low muscle strength and to evaluate physical activity and sleep characteristics in people with type 2 diabetes in Kuwait. Additionally, equivalent data from the UK Biobank cohort were compared”

The corrected sentence appears below:

“This study demonstrates that insufficient sleep, physical inactivity, and muscle weakness are prevalent in people with type 2 diabetes, especially in Kuwait. Importantly, these observations warrant urgent and effective interventions to improve sleep, muscle strength, and physical activity, especially in Kuwait.”

The authors apologize for these errors and state that they do not change the scientific conclusions of the article in any way. The original article has been updated.

